# Hospital admission and vaccination as predictive factors of long COVID-19 symptoms

**DOI:** 10.3389/fmed.2022.1016013

**Published:** 2022-11-11

**Authors:** Esperanza Romero-Rodríguez, Luis Ángel Pérula-de Torres, Rafael Castro-Jiménez, Jesús González-Lama, Celia Jiménez-García, Jerónimo J. González-Bernal, Josefa González-Santos, Rodrigo Vélez-Santamaría, Esteban Sánchez-González, Mirian Santamaría-Peláez

**Affiliations:** ^1^Maimonides Biomedical Research Institute of Cordoba (IMIBIC), Reina Sofia University Hospital, University of Cordoba, Cordoba, Spain; ^2^Multiprofessional Teaching Unit of Family and Community Care, Córdoba and Guadalquivir Health District, Córdoba, Spain; ^3^Córdoba and Guadalquivir Health District, Córdoba, Spain; ^4^Reina Sofía University Hospital, Córdoba, Spain; ^5^Health Center “Matrona Antonia Mesa Fernández”, Cabra Clinical Management Unit, AGS Sur de Córdoba, Córdoba, Spain; ^6^Department of Health Sciences, University of Burgos, Burgos, Spain; ^7^Department of Medicine, Central University of Catalonia, Barcelona, Spain

**Keywords:** long COVID, persistent COVID, COVID-19, symptoms, risk factor, admission, post-COVID

## Abstract

**Background:**

Since the beginning of the COVID-19 pandemic, a great variability of symptoms that affect all organs and systems of the body has been identified in patients with SARS-CoV-2 infection; this symptomatology can sometimes persist over time, giving rise to the so-called long COVID or post-COVID. The aim of this study is to delve into the clinical characterization of these patients, as well as to take into account the influence of factors such as hospitalization, admission to ICU, history of pneumonia, or vaccination status on the persistence of symptoms.

**Material and methods:**

An observational, descriptive, multicenter, and retrospective study was designed with a series of cases of people who presented long COVID, which includes univariate, bivariate, and multivariate analyses. Data were obtained from an online *ad hoc* questionnaire, and statistical analysis was performed using SPSS Software Version 25 (IBM-Inc., Chicago, IL, USA).

**Results:**

Hospitalization, ICU admission, history of pneumonia, and vaccination were predictive factors (positive or negative) for the following long-COVID symptoms: headache, menstrual disorders, joint pain, cough, chills, nasal congestion, back pain, abdominal pain, weight loss, eye discomfort, facial erythema, itching, tremors, dizziness, seizures, sleeping difficulty, dry eyes, palpitations, fatigue, paresthesia, dyspnea, aphonia, chest pain, high blood pressure, vomiting, memory loss, brain fog, hypothermia, low blood pressure, sputum or phlegm, lack of concentration, hair loss, and erectile dysfunction.

**Conclusion:**

This study provides evidence on the clinical characterization of patients suffering from long COVID in order to offer them the most appropriate treatments.

## Introduction

The SARS-CoV-2 infection presents associated symptomatology of great interest to public health due to both the transmission and the appearance of new variants rapidly on all continents. The COVID-19 disease manifests itself in various ways; it can be asymptomatic, mild, moderate, or severe (1). There is a great variability of symptoms presented in patients with SARS-CoV-2 infection, as this virus can affect all organs and body systems (2). The World Health Organization (WHO) initially identified fever, cough, or asthenia as possible symptoms of the SARS-CoV-2 infection; however, subsequent studies suggested additional symptoms associated with this infection, such as olfactory and taste dysfunction, coagulation alterations, or gastrointestinal symptoms (nausea or abdominal pain) (3).

A variety of Spanish studies (4, 5), as well as some international studies (6, 7), addressed the clinical characteristics of the COVID-19 disease. Fever, dry cough, tiredness or fatigue, dyspnea, pharyngeal pain, chills, and diarrhea are the symptoms that appear most frequently in the acute phase of the disease, but the symptomatology can be prolonged over time, leading to persistent COVID-19. The definition of this condition has changed throughout the COVID-19 pandemic (8) and has acquired different denominations, such as long COVID, post-acute COVID-19, post-acute sequelae of SARS CoV-2 infection (PASC), long-term effects of COVID-19, or chronic COVID-19.

The prevalence of persistent COVID-19 is around 10%, although it ranges between 10 and 36% depending on the sociodemographic and clinical characteristics of the study population (9–11). A study conducted in Denmark (12) showed a prevalence of 36% in the Danish population diagnosed with COVID-19 disease. Similarly, research conducted in the United Kingdom (13) revealed that 13% of the population presented persistent symptoms of COVID-19 and those who had needed hospital care during the acute phase of the infection were more likely to present long COVID. Similarly, a German study (14) highlighted that persistent COVID-19 can occur even after the acute phase with very mild symptoms that have been treated on an outpatient basis. Similarly, in Spain, the survey conducted by the Spanish Society of General Medicine (15) estimated the prevalence of persistent COVID-19 at around 15% and described the general characteristics of patients with persistent COVID-19 who were diagnosed during the first wave of the pandemic, highlighting that factors such as age (50% between 36 and 50 years), sex (more frequent in women), or frequent symptomatology (asthenia, malaise, headache, or mood disturbance) were associated with persistent COVID-19 symptoms.

Initially, the WHO identified 33 symptoms of long COVID (16) although subsequent scientific evidence suggested more symptoms associated with the persistent situation of COVID-19 (17). Raveendran, Jayadevan, and Sashidharan (18) detected several symptoms of long COVID, such as fatigue, cough, chest tightness, respiratory distress, palpitations, myalgia, and concentration difficulty. Aiyegbusi et al. (19) analyzed both physical and mental symptoms and found that the top ten most prevalent symptoms were fatigue, shortness of breath, muscle pain, joint pain, headache, cough, chest pain, altered smell, altered taste, and diarrhea, although they also indicated that there are other common symptoms such as cognitive impairment, memory loss, anxiety, and sleep disorders; Beyond the symptoms, they determined that people affected by long COVID frequently suffer a worsening of their life quality and even problems related to the work sphere. The presence of more than five symptoms throughout the 1st week of the SARS-CoV-2 infection has been described as a risk factor for long COVID. The symptoms developed in that 1st week that were considered more predictive for long COVID were fatigue, headache, dyspnea, hoarseness, and myalgia, for both sexes (13). However, another study ruled out patients with only moderate symptoms, those with a normal chest X-ray, and those with frailty (13).

Since the appearance of the first cases of infection by the SARS-CoV-2 virus, new data on its clinical and epidemiological characteristics have been incorporated; however, there is less scientific evidence that addresses how certain characteristics such as hospital admission, history of pneumonia, or vaccination can influence the appearance of different symptoms from the clinical and epidemiological point of view of persistent COVID-19.

The available evidence on COVID-19 is mainly focused on hospitalized cohorts (20–24) that address the factors that are associated with the development or not of persistent COVID-19, including advanced age (over 60 years), female sex, the presence of comorbidities, the existence of multiple symptoms during the acute phase, or admission to intensive care. Another study performed with patients who had been previously hospitalized identified three clusters: a group of patients with fewer medical comorbidities, fewer COVID-19 symptoms in the acute phase, fewer post-COVID symptoms and no functional symptoms, and two groups of patients with a higher number of medical comorbidities, more symptoms of COVID-19 in the acute phase, increased number of post-COVID symptoms, and more limitations in activities of daily living (25). In non-hospitalized patients, age, female sex, belonging to a minority ethnicity, socioeconomic deprivation, smoking, obesity, and a wide range of comorbidities are risk factors for developing long COVID (17). In contrast, another study indicated that long COVID was not directly attributed to the effects of acute COVID-19 infection or its severity and posits that the biopsychosocial effects of COVID-19 could play a more important role in its etiology (17).

The WHO and the Long Covid Forum Group established a research priority on long COVID to improve the clinical characterization of these patients to offer them appropriate treatments (26). Crook et al. (2) exposed the mechanisms and risk factors of long COVID, in addition to a treatment proposal depending on the symptomatology that appears in each case, for which it would be useful to know the predictors of the different symptoms. However, there is less evidence on this, so studies are needed to identify the clinical characteristics and predictors for the symptomatology of persistent COVID-19.

Therefore, considering all of the abovementioned points, this study aimed to analyze whether hospital admission, ICU admission, history of pneumonia, and vaccination can be predictor factors for the different symptoms of persistent COVID-19 or long COVID.

## Materials and methods

### Study design

An observational, descriptive, multicenter, and retrospective study was designed with a series of cases of people who presented long COVID or post-COVID syndrome, understanding that the “condition occurs in individuals with a history of probable or confirmed SARS-CoV-2 infection, usually 3 months from the onset, with symptoms that last for at least 2 months and cannot be explained by an alternative diagnosis” (9). The study was conducted in the South Healthcare Management Area of Córdoba and the Cordoba and Guadalquivir Healthcare District from July 2021 to July 2022.

### Study population

The study included patients from the general adult population who attended the Spanish National Health System with the following selection criteria: (a) Should be a resident of Spain and have an age ≥14 years, (b) should meet the clinical-epidemiological criteria of the long COVID-19 disease, and (c) should consent to participate in the research study.

As for sample size, there is less knowledge about the actual prevalence of long COVID-19. Assuming a long COVID-19 prevalence of 10.0% (11), a sample of 138 individuals would be enough for a confidence level of 95% and an accuracy of ±5% units (calculations made with the Granmo program: https://www.imim.es/ofertadeserveis/software-public/granmo/).

The research project obtained the authorization of the Direction/Management of the Córdoba and Guadalquivir Primary Care Health District and the South Health Area of Córdoba, as well as the approval of the Clinical Research Ethics Committee of the Reina Sofía Hospital in Córdoba (reference: 5033). Informed consent was requested as part of the online questionnaire, which gives voluntariness to the study patients. The processing of the data was in accordance with the provisions of the European Data Protection Regulation and Organic Law 3/2018 on the Protection of Personal Data and the guarantee of digital rights.

### Instruments

The information about the participating patients was obtained from an online questionnaire (https://docs.google.com/forms/d/e/1FAIpQLSeO2odLrsCGf_aA6PbRbAziMA3ZP43wAmo81rRgKuLmmnaXCg/viewform), which was forwarded to the partners of the persistent COVID associations existing in Spain. The questionnaire was designed and approved by members of the Multiprofessional Teaching Unit of Family and Community Care of the Córdoba and Guadalquivir Health District, with proven experience in the design and validation of surveys. The questionnaire was subjected to a process of consensus, apparent logic, and content validation (face validity).

### Variables

Information about sociodemographic variables (age, sex, residential area, and employment status), as well as the following personal health history, namely, the vaccination status for COVID-19 and symptoms presented in patients with long COVID-19, was collected. Hospital and intensive care unit (ICU) admissions, as well as history of pneumonia, were taken into consideration as background. Regarding the long COVID-19 symptoms, a list of 56 possible symptoms was taken into account for the situation of persistent COVID-19: sore throat, headache, joint pain, muscle pain, unusual tiredness or fatigue, breathing difficulty, lack or loss of smell, lack or loss of taste, cough, dyspnea, fever, sweating, chills or shivering, nasal congestion, aphonia or hoarseness, malaise, chest pain, back pain, chest tightness, diarrhea, stomach pain, abdominal pain, vomiting, nausea, loss of appetite, weight loss, hypothermia, eye discomfort (conjunctivitis, dry eye, blurred vision, foreign body, and congestion), facial erythema, limbs pseudo freezing (Acro syndrome, chilblain-like lesions), sputum or phlegm (bronchial discharge), hemoptysis (bloody sputum), swelling or inflammation of the fingers, itching (pruritus), hives or eczema on the skin (rashes), tremors, dizziness, seizures, memory loss, mental confusion, sleeping difficulty, lack of concentration/attention deficit, mental fog, post-traumatic stress, paresthesia, swallowing difficulty, ear beeps or tinnitus, dry eyes, conjunctivitis, palpitations, high blood pressure, low blood pressure, hair loss, erectile dysfunction (men), and menstrual disorders (women).

### Statistical analysis

The participants were asked to fill in the online questionnaire on Google Drive. Later, the responses were exported to an Excel sheet from Google Drive and statistically treated with the SPSS Software Version 25 (IBM-Inc., Chicago, IL, USA).

First, a descriptive analysis of the studied variables was performed using frequencies and percentages when they were qualitative or categorical variables and measures of central tendency, dispersion, and position when they were quantitative variables. We estimated confidence intervals for 95% (95% CI) for safety of the main parameters.

Next, a bivariate analysis (chi-square test) was performed, considering the exact bilateral significance (Fisher's exact test) as there are 2 × 2 tables in all cases, to determine if there was any relationship between each of the 56 persistent symptoms of long COVID and hospital admissions or vaccinations. Once the significant relationships were defined, an analysis of the magnitude of the effect of the association was also performed, calculating the odds ratio (OR, since all the variables were dichotomous) to determine if the variables were predictors for the symptoms with which they had previously demonstrated a relationship. For this analysis, an OR < 1.68 is considered to be of negligible magnitude, an OR between 1.68 and 3.47 was considered small, an OR between 3.47 and 6.71 was considered moderate, and an OR > 6.71 was considered large (27), so magnitudes below 1.68 were not taken into account as they were not considered clinically relevant.

Finally, once determined by the previous bivariate analysis, the variables were presumably related in a bivariate way to hospital admissions or vaccinations, and to establish a predictive model that included the factors that had shown the predictive capacity for the symptoms of long COVID, a binary logistic regression analysis was performed (step-by-step method, backward, with a reason of plausibility) to be able to control the predictor and/or confounding factors. The dependent variables were each of the symptoms with more than one predictive factor, and the independent variables were each of those factors to obtain the coefficient β, the statistician χ2 Wald, *p*-value, and RO = e[βi^*^ (±Δi)] adjusted with their 95% confidence interval limits. For the analysis of statistical significance, a *p*-value < 0.05 was established. The goodness-of-fit of the model was analyzed using the Hosmer-Lemeshow test.

## Results

The total number of participants with long COVID was 681. The mean age was 45.78±9.65 (SD), ranging from 14 to 76 years (mean 95% CI: 46.02–46.46). Of 681 participants, women made up 83.1%; 79.3% resided in urban areas (>20,000 inhabitants); and 41.0% were on sick leave from work due to persistent COVID-19. Of the total patients, 23.3% were hospitalized, with 3.5% in an ICU, 29.8% presenting pneumonia after the diagnosis of COVID-19, and 87.4% being vaccinated against COVID-19.

To find out if the variables hospitalization, ICU admission, and history of pneumonia were related to each of the persistent symptoms of long COVID, a chi-square test was performed. The significant results obtained in these analyses are shown in Tables 1, 2; the rest of the symptoms showed no relationship.

**Table 1:** Relationship between long COVID symptoms and hospitalization, ICU admission or pneumonia.

	**Hospitalization**		**Admitted to ICU**		**Pneumonia**	
	**Value**	***p*-value**	**Value**	***p*-value**	**Value**	***p*-value**
Headache	–	–	4.382	0.043	–	–
Muscle pain	4.737	0.030	–	–	–	–
Fatigue	4.364	0.035	–	–	–	–
Cough	–	–	–	–	4.918	0.028
Dyspnoea	10.162	0.001	–	–	6.654	0.010
Aphonia	7.533	0.008	–	–	7.579	0.008
Malaise	4.206	0.044	–	–	–	–
Chest pain	10.503	0.001	–	–	5.889	0.018
Back pain	4.616	0.035	–	–	–	–
Chest tightness	–	–	–	–	4.963	0.029
Diarrhea	–	–	4.431	0.035	–	–
Stomach pain	6.361	0.014	4.019	0.045	–	–
Vomiting	–	–	–	–	6.350	0.019
Hypothermia	–	–	–	–	4.073	0.045
Sputa	–	–	–	–	12.255	0.001
Memory loss	–	–	–	–	9.622	0.001
Mental confusion	6.631	0.011	–	–	9.914	0.002
Difficulty sleeping	4.886	0.030	–	–	4.439	0.037
Lack of concentration	4.794	0.033	–	–	8.356	0.004
Brain fog	4.152	0.049	–	–	7.619	0.006
Post–traumatic stress	6.860	0.011	–	–	6.695	0.012
Paraesthesia	6.275	0.015	–	–	6.070	0.016
High blood pressure	13.101	<0.001	–	–	6.121	0.017
Low blood pressure	4.653	0.032	–	–	4.879	0.026
Hair loss	–	–	6.167	0.016	–	–
Erectile dysfunction	15.309	<0.001	42.736	<0.001	5.573	0.030
Menstrual disorders	–	–	6.155	0.009	–	–

ICU, intensive care unit.

**Table 2:** Relationship between long COVID symptoms and vaccination.

	**Vaccinated**	
	**Value**	***p*-value**
Joint pain	4.877	0.034
Fatigue	6.030	0.010
Cough	7.398	0.009
Chills	10.558	0.002
Nasal congestion	4.812	0.033
Back pain	4.342	0.045
Abdominal pain	5.703	0.024
Weight loss	6.349	0.020
Hypothermia	12.930	0.001
Eye discomfort	15.535	<0.001
Facial erythema	9.403	0.004
Sputa	21.308	<0.001
Itching	6.215	0.010
Tremors	5.690	0.014
Dizziness	5.285	0.025
Dizziness	7.074	0.011
Seizures	5.931	0.027
Difficulty sleeping	8.301	0.004
Lack of concentration	4.208	0.042
Paraesthesia	5.516	0.022
Dry eyes	18.538	<0.001
Palpitations	9.663	0.002
Hair loss	16.627	<0.001

Tables 3–6 show how hospital admission, ICU admission, history of pneumonia, and vaccination appear as predictive factors for long-COVID symptoms. Hospital admission is a positive predictor for several symptoms (OR 1.58–3.77) but only a negative predictor for low blood pressure (OR 0.51). ICU admission is a positive predictor for erectile dysfunction (OR 12.38) and a negative predictor for headache, hair loss, and menstrual disorders (OR 0.11–0.42). The history of pneumonia appears to be a positive predictor also for several symptoms (OR 1.69–2.28) and a negative predictor for hypothermia (OR 0.66) and low blood pressure (OR 0.54). Vaccination is a negative predictor for all the significant symptoms (OR 0.19–0.60).

**Table 3:** Admission to a hospital – symptoms of long COVID.

**Symptoms**	**OR**	**OR limits CI 95%**	
		**Lower**	**Upper**
Fatigue	2.13	1.03	4.40
Dyspnea	1.93	1.28	2.90
Aphonia	1.70	1.16	2.49
Chest pain	1.80	1.26	2.58
Mental confusion	1.70	1.13	2.55
Difficulty concentrating	1.71	1.05	2.79
Paresthesias	1.58	1.10	2.26
Elevated blood pressure	2.09	1.39	3.14
Low blood pressure	0.51	0.27	0.94
Erectile dysfunction	3.77	1.86	7.97

OR, Odds Ratio.

**Table 4:** Admission to ICU – symptoms of long COVID.

**Symptoms**	**OR**	**OR limits CI 95%**	
		**Lower**	**Lower**
Headache	0.42	0.18	0.96
Hair loss	0.24	0.07	0.81
Menstrual disorders	0.11	0.01	0.88
Erectile dysfunction	12.38	4.84	31.63

OR, Odds Ratio.

**Table 5:** Presence of pneumonia after COVID-19 diagnosis – symptoms of long COVID.

**Symptoms**	**OR**	**OR limits CI 95%**	
		**Lower**	**Upper**
Vomiting	2.24	1.18	4.27
Sputa	2.16	1.39	3.34
Memory leak	1.83	1.24	2.70
Mental confusion	1.81	1.24	2.64
Lack of concentration	1.93	1.23	3.03
Brain fog	1.69	1.16	2.46
Hypothermia	0.66	0.45	0.99
Low blood pressure	0.54	0.31	0.94
Erectile dysfunction	2.28	1.13	4.62

OR, Odds Ratio.

**Table 6:** Vaccination – symptoms of long COVID.

**Symptoms**	**OR**	**OR limits CI 95%**	
		**Lower**	**Upper**
Joint pain	0.51	0.28	0.93
Fatigue	0.19	0.48	0.82
Cough	0.52	0.33	0.84
Chills	0.46	0.29	0.74
Nasal congestion	0.58	0.35	0.94
Back pain	0.60	0.37	0.97
Abdominal pain	0.56	0.35	0.90
Weight loss	0.49	0.28	0.86
Hypothermia	0.42	0.26	0.68
Eye discomfort	0.38	0.23	0.62
Facial erythema	0.40	0.22	0.73
Sputa	0.30	0.18	0.51
Itching	0.54	0.34	0.88
Tremors	0.55	0.33	0.90
Dizziness	0.57	0.35	0.92
Dizziness	0.53	0.33	0.85
Seizures	0.31	0.11	0.83
Difficulty sleeping	0.46	0.26	0.78
Lack of concentration	0.49	0.24	0.98
Paresthesias	0.57	0.36	0.91
Dry eyes	0.36	0.22	0.58
Palpitations	0.47	0.29	0.76
Low blood pressure	0.54	0.30	0.98
Hair loss	0.38	0.24	0.61

OR, Odds Ratio.

Table 7 shows the results of the backward stepwise regression model after the elimination of the variables that, although they were significant in the previous analysis, finally did not contribute anything to the model in each case.

**Table 7:** Binary logistic regression: long COVID symptoms – significant OR variables.

	**R2 of Nagelkerke**	**β**	**Standard error**	**Wald**	***p*-value**	**OR (CI 95%)**
**Fatigue (Hosmer-Lemeshow: 0.746)**						
Admission hospital	0.045	0.90	0.389	5.37	0.020	2.46 (1.15–5.29)
Vaccination		−1.65	0.729	5.14	0.023	0.19 (0.04–0.79)
Constant		−2.82	0.364	60.31	<0.001	0.05
**Hypothermia (Hosmer-Lemeshow: 0.682)**						
Pneumonia	0.035	−0.42	0.205	4.19	0.041	0.65 (0.44–0.98)
Vaccination		−0.85	0.244	12.38	<0.001	0.42 (0.26–0.68)
Constant		−1.47	0.182	65.90	<0.001	4.36
**Sputa (Hosmer-Lemeshow: 0.146)**						
Pneumonía	0.078	0.82	0.229	13.04	<0.001	2.28 (1.45–3.57)
Vaccination		−1.22	0.273	20.16	<0.001	0.29 (0.17–0.50)
Constant		1.44	0.181	63.84	<0.001	4.25
**Lack of concentration (Hosmer-Lemeshow: 0.656)**						
Pneumonia	0.33	0.68	0.234	8.64	0.003	1.99 (1.25–3.15)
Vaccination		−0.72	0.353	4.14	0.042	0.48 (0.24–0.97)
Constant		−1.77	0.209	72.21	<0.001	0.16
**Paresthesias (Hosmer-Lemeshow: 0.642)**						
Admission hospital	0.024	0.48	0.186	6.75	0.009	1.62 (1.12–2.33)
Vaccination		−0.57	0.237	5.90	0.015	0.56 (0.35–0.89)
Constant		0.16	0.163	0.98	0.321	1.17
**Low blood pressure (Hosmer-Lemeshow: 0.912)**						
Pmeumonia	0.23	−0.59	0.279	4.49	0.034	0.55 (0.30–0.099)
Vaccination		−0.60	0.301	3.97	0.046	0.54 (0.32–0.095)
Constant		2.39	0.254	89.43	<0.001	10.99
**Erectile dysfunction (Hosmer-Lemeshow: 0.938)**						
Admission hospital	0.11	0.83	0.421	3.90	0.048	2.29 (1.01–5.24)
ICU		1.94	0.545	12.74	<0.001	6.99 (2.40–20.36)
Constant		0.66	0.434	2.33	0.126	1.94

ICU, Admission to Intensive Care Unit.

This last step of logistic regression has excluded hospitalization for the lack of concentration and low blood pressure and history of pneumonia for erectile dysfunction from the explanatory models. In the case of mental confusion and hair loss, the Hosmer-Lemeshow test indicates that this model has not got an appropriate fit (χ^2^ < 0.001).

## Discussion

The present study aimed to analyze whether hospital admission, ICU admission, history of pneumonia, and/or vaccination can be predictors of the different symptoms of persistent COVID-19 or long COVID.

Our results showed that hospitalization, ICU admission, history of pneumonia, and being vaccinated against COVID-19 were predictive factors (positive or negative) for headache, menstrual disorders, joint pain, cough, chills, nasal congestion, back pain, abdominal pain, weight loss, eye discomfort, facial erythema, itching, tremors, dizziness, seizures, sleeping difficulty, dry eyes, palpitations, fatigue, paresthesia, dyspnea, aphonia, chest pain and high blood pressure, vomiting, memory loss, brain fog, hypothermia, low blood pressure, sputum or phlegm, lack of concentration, hair loss, and erectile dysfunction. Nowadays, there is less evidence that focuses on the predictive factors for each of the symptoms. However, some investigations determined that patients who required hospitalization present a significant proportion of late clinical events and persistent symptoms in the medium-term (2 months) and the long-term (6 months) (28).

Fernandez-de-las-Peñas et al. found that a higher symptom burden in the acute phase of COVID-19 infection and a higher number of preexisting medical comorbidities may predict a higher likelihood of persistent COVID symptoms, particularly fatigue or dyspnea 3–6 months later. These factors are also mentioned in other recent studies, suggesting that post-COVID symptoms are more prevalent in patients with severe symptoms at the onset of infection and in hospitalized patients (2, 28, 29), which, in turn, is related to the need for ICU admission (30). ICU admission is also established as a risk factor for long COVID (31) and a longer length of hospital stay, which, in turn, has also been indicated as a factor associated with persistence at 6-month follow-up (30). In the same vein, a shorter hospital stay was inversely associated with prolonged COVID syndrome (29), as well as a greater number of symptoms during the acute stage of the process (2, 31–33). Thus, patients who required ICU admission reported a greater decrease in the quality of life than those who did not (34). In contrast, studies also found that the severity of acute infection is not a risk factor for long COVID (32).

Our results showed that hospitalization is a risk factor for fatigue and paresthesia (symptoms for which vaccination is also a protective factor), as well as dyspnea, aphonia, chest pain, and high blood pressure. One study compared post-COVID-19 fatigue with chronic fatigue syndrome as there are overlaps between them; however, although fatigue is shown to be an important symptom of long COVID, research showed that there is no relationship between COVID-19 severity and long-term fatigue (2). In addition, dyspnea is a common symptom of long COVID that could be associated with people at high risk of developing respiratory distress (older adults, people with previous respiratory pathology or with prolonged hospital stays) (2, 29). In our study, only hospitalization was found to be a risk factor for the persistent symptom of dyspnea; however, our results showed that having suffered from pneumonia is also a risk factor for other symptoms that may be related to respiratory distress or respiratory symptoms such as sputum or phlegm; although it should be noted that vaccination was a protective factor for these symptoms.

Regarding the symptoms of patients who required ICU admission, ICU admission itself proved to be a protective factor for headache, menstrual disorders, and hair loss (as well as being vaccinated for the latter); however, it became a risk factor for erectile dysfunction, along with those who were hospitalized. Garrigues et al. analyzed 120 patients and identified that the most common persistent symptoms in a sample of hospitalized people, at 110 days on average after admission, were fatigue (55%), dyspnea (42%), memory loss (34%), concentration disorders (28%), and sleep disturbances (30.8%). In contrast, there was no difference between “standard patients” and those who needed ICU during their hospitalization (35) for these symptoms. This study points to hospitalization itself as a risk factor for dyspnea, pneumonia, memory loss, and poor concentration, but not ICU admission.

Furthermore, studies showed that physical, cognitive, and mental deterioration, which persists long after the acute COVID-19 disease, is common in ICU survivors (36) and that ICU admission is a risk factor for long COVID. In contrast, other researchers reported that there is no difference in the proportion of patients with persistent symptoms between those with and without ICU admission; however, ICU patients showed exertional dyspnea and asthenia more frequently (36). Significant differences in symptom persistence have also been found in anosmia/dysgeusia, such that there is a higher incidence of them in patients with mild disease, while there is a higher incidence of fatigue, dyspnea, neurological disorders, and rheumatological symptoms in patients with severe disease (31).

However, vaccination is a protective factor for other symptoms such as joint pain, cough, chills, nasal congestion, back pain, abdominal pain, weight loss, eye discomfort, facial erythema, itching, tremors, dizziness, convulsions, difficulty sleeping, dry eyes, and palpitations. Some studies pointed in the same direction as ours, so vaccination is useful for improving symptoms in people who already have the disease (37–39). Many of these investigations found no difference in the type of vaccine used (37, 38, 40), while others found greater improvement in those who received mRNA vaccines compared to adenoviral vector vaccines (39). For every symptom, Moderna had a more positive impact and was more beneficial than adenoviruses for fatigue, mental confusion, myalgia, gastrointestinal symptoms, and autonomic dysfunction (39). In contrast, there is no evidence associating long COVID with an increased incidence of adverse effects after vaccination (41). Insomnia is also commonly reported after recovery from COVID-19, but stress, anxiety, and other negative emotions stemming from the pandemic are also associated with sleep problems, so it is unclear whether the sleep disturbances are due to the infection itself, to the negative effects of the pandemic, or to a combination of both (2).

In addition, vaccines are an important preventive strategy for long COVID (40, 42), as they can very effectively prevent severe infections and hospital admissions, which are risk factors for long COVID (43). The likelihood of experiencing symptoms beyond 28 days post-infection is reduced by 50% with the full vaccination schedule. This regimen also reduces the likelihood of having more than five symptoms in the 1st week of infection by 31% and reduces the likelihood of hospitalization by 71%, so the likelihood of developing long COVID would be similarly decreased by reducing both risk factors (32). The improvement in symptoms of long COVID after vaccination has not been explained yet, although it is theorized that vaccination counteracts immune dysregulation associated with symptomatic persistence (40). Finally, pneumonia is a risk factor for symptoms such as vomiting, memory loss, and brain fog, although both history of pneumonia and vaccination are protective factors for hypothermia and low blood pressure. Brain fog is a common and debilitating symptom in long COVID and, in this study, having developed pneumonia after a SARS-CoV-2 infection is a risk factor for brain fog as a persistent symptom. One study also reports a high prevalence of both physical and mental health symptoms in patients who have been treated in hospitals after pneumonia, although it is notable from its results that the severity of pneumonia is not a good predictor of long-COVID symptoms (44).

The explanation of symptoms with more than one predictor is based on logistic regression analysis, except for mental confusion and hair loss, where it is based on OR, as the logistic regression model does not fit adequately.

As for limitations, it is important to note that the type of survey used may introduce a kind of selection bias. In addition, there is also a possible information bias as the information came from the patient himself and was not contrasted or confirmed by a physician, so the results should be taken into account with caution. Furthermore, although there is evidence on the predictors of long COVID as a whole, the evidence on the predictors of each of the symptoms is very scarce, so in this part, there has been a limitation in contrasting the results obtained with other similar studies; however, the scarcity of similar studies is a strong point to be taken into account in this research and it is advisable to continue to deepen this line of research. This research is presented as a case series study without a control group, which implies a methodological limitation that could compromise its validity. Therefore, the results obtained must be taken into account together with the limitations of the type of study design, and further research is needed to contrast the results offered. Another limitation of this study is the lack of temporality of the COVID-19 infection and the administration of the COVID-19 vaccine. Future studies should evaluate the chronological association between both variables.

In conclusion, hospital admission, ICU admission, history of pneumonia, and vaccination are predictors of some of the symptoms of persistent COVID-19 or long COVID. This provides evidence for the priority set by the WHO and the Long Covid Forum Group to improve the clinical characterization of patients suffering from long COVID in order to offer them the most appropriate treatments. Therefore, this study contributes to identifying predictors of the different symptoms that may appear in the course of long COVID, which is important to be able to develop preventive and symptomatic treatments early and to plan recovery interventions.

## Data availability statement

The raw data supporting the conclusions of this article will be made available by the authors, without undue reservation.

## Ethics statement

The studies involving human participants were reviewed and approved by Comité de Ética en Investigación Clínica Hospital Reina Sofía, Córdoba, Spain. The patients/participants provided their written informed consent to participate in this study.

## Author contributions

ER-R, LP-dT, RC-J, JG-L, CJ-G, JG-B, JG-S, RV-S, ES-G, and MS-P were involved in the conception and design of the study, as well as the data acquisition. JG-B and MS-P performed the analyses. ER-R, MS-P, and LP-dT drafted the manuscript, which was revised by JG-S, RV-S, and ES-G. All authors listed have made a substantial, direct, and intellectual contribution to the study and have approved it for publication.

## Funding

This research was funded by the call for research and innovation projects in the field of primary care, regional hospitals, and high-resolution hospital centers of the Public Health System of Andalusia in 2021 by the Progreso y Salud Foundation of the Ministry of Health and Families of the Junta de Andalucía, with EXP. No. AP-0184-2021- C2-F2.

## Conflict of interest

The authors declare that the research was conducted in the absence of any commercial or financial relationships that could be construed as a potential conflict of interest.

## Publisher's note

All claims expressed in this article are solely those of the authors and do not necessarily represent those of their affiliated organizations, or those of the publisher, the editors and the reviewers. Any product that may be evaluated in this article, or claim that may be made by its manufacturer, is not guaranteed or endorsed by the publisher.
